# The Effectiveness of a Dark Septate Endophytic Fungus, *Cladophialophora chaetospira* SK51, to Mitigate Strawberry Fusarium Wilt Disease and With Growth Promotion Activities

**DOI:** 10.3389/fmicb.2020.00585

**Published:** 2020-04-15

**Authors:** Wiwiek Harsonowati, Malek Marian, Surono, Kazuhiko Narisawa

**Affiliations:** ^1^Department of Symbiotic Science of Environment and Natural Resources, United Graduate School of Agriculture Science, Tokyo University of Agriculture and Technology, Tokyo, Japan; ^2^Department of Bioresource Science, College of Agriculture, Ibaraki University, Ibaraki, Japan; ^3^Department Soil Biology and Health, Indonesian Agency for Agricultural Research and Development, Jakarta, Indonesia

**Keywords:** Fusarium wilt, dark septate endophytes, *Cladophialophora chaetospira*, flower bud initiation, fruit formation, growth promotion

## Abstract

Strawberry Fusarium wilt, caused by the virulent fungus *Fusarium oxysporum formae speciales fragariae* (Fof) is a devastating soil-borne disease that causes severe production losses worldwide, including Japan. Fof is one of the top 10 fungal pathogens that threaten global crop security, and a method to effectively control this pathogen has yet to be found. This study aimed to investigate the effectiveness of dark septate endophytic (DSE) fungi against Fof to develop an efficient, effective, and environmentally friendly approach to improve plant health and fitness. A total of 19 fungal isolates were assessed, out of which three (SK47, SK48, and SK51) were selected based on their effectiveness in disease suppression in controlled growth chamber conditions using a soil system. Isolates SK47, SK48, and SK51 suppressed disease severity by 85.71, 61.90, and 90.48%, respectively. Molecular identification based on highly conserved small subunit (SSU), internal transcribed spacer (ITS), and large subunit (LSU) nrRNA regions identified these isolates as DSE *Exophiala* sp., *Exophiala pisciphila*, and *Cladophialophora chaetospira*, respectively. The sequences were deposited under accession numbers MN811693–MN811695 in the GenBank database. Notably, our results revealed that isolate *C. chaetospira* SK51 possessed superior growth promotion activities as well as disease suppression by significantly increased plant growth parameters (shoot and root dry mass, chlorophyll content, flower bud initiation, and number of fruit) in comparison to control plants and other two fungal candidates. Root colonization by *C. chaetospira* SK51 was visualized, and it was confirmed that the symbiosis with strawberry plants occurred successfully. Our results provide new insights in the application of DSE fungus *C. chaetospira* SK51 as a biocontrol agent on strawberry plants could promote plant growth, flower bud initiation, and fruit formation. *C. chaetospira* SK51 exhibited remarkable beneficial traits for the host plant, and it can potentially be applied in the development of new, safe, and effective treatments as an alternative to chemical fertilizers and fungicides for sustainable crop protection.

## Introduction

Strawberries are one of the most important fruit crops grown worldwide (Husaini and Abdin, 2008). Strawberry production has shown a remarkable increase of about 36.7% and an expansion in area of about 13.7% in the period between 2007 and 2017 (FAO, 2019). Japan is one of the leading strawberry producers (9th in the world) with fruit production of 158,702 tons/year in 2017 from a total harvested area of 5,312 ha (FAO, 2019). Plant diseases represent major challenges to strawberry production, causing damage, economic loss in nursery, and field production around the world including Japan (Okayama, 1991; Tezuka and Makino, 1991; Mori and Kitamura, 2003). *Fusarium oxysporum* is a host-specific fungal pathogen, which infects diverse plants includes both dicots (e.g., bean, carnation, and tomato), and monocots (e.g., banana, orchids, and palms). The host specificity of pathogenic strains has led to the concept of *formae speciales* (f. sp.) with total 106 well-characterized f. sp., and 37 insufficiently documented ones. Among these, *Fusarium oxysporium* f. sp. *fragariae* (Fof) is the specific pathogen responsible for the wilt of strawberry disease (Winks and Williams, 1965). Fof is one of the most devastating soil-borne fungal pathogens that affects strawberry field production in Japan; in Akita Prefecture in Northern Japan (Mori and Kitamura, 2003; Takahashi et al., 2003) up to 50% of plants are infected by Fof (Fang et al., 2011). Variation in susceptibility to Fof has been reported for Japanese strawberry cultivars (Paynter et al., 2014).

Typically, Fof penetrates strawberry plants through the roots, severely affecting the roots and crowns, resulting in rapid wilting and eventually death of the plants (Koike et al., 2009; Fang et al., 2011). Fof usually involves a vascular wilt syndrome, as a result of the xylem vessels becoming blocked (Leslie and Summerell, 2006). In addition, transplants are infected through runners from infected mother plants (Matuo et al., 1980). Fof is difficult to eradicate and can remain viable in the soil as resting spores (chlamydospores) for many years before these spores are stimulated to grow (Koike and Gordon, 2015).

Current management strategies used against Fof include disease-resistant cultivars, soil solarization, and chemical soil fumigant. However, breeding programs for developing new disease-resistant cultivars to manage Fof have several limitations such as cultivars varying in their level of resistance depending on the environmental conditions under which the plants are grown, and differences in isolate virulence may result in varied disease incidence (Kodama, 1974; Fang and Barbetti, 2014). Soil solarization is a culture control method for Fof that has been rapidly introduced to strawberry cultivation (Kodama and Fukui, 1979). However, the implementation of this technique in Japan is limited for plastic greenhouses in the summer season when the air temperature is high and soil temperatures range from 50 to 60°C (Kodama and Fukui, 1979). Strawberry production has relied on the use of several chemicals for soil fumigation, for example, 1,3-dichloropropene plus chloropicrin, dazomet, and metam sodium (Dominguez et al., 2014). Growers often apply pesticides 8–10 times or even more during a cropping season, hence disease control is a major expense in strawberry production (Murthy and Pramanick, 2012). Therefore, developing other alternative or supplementary approaches are required. Biological control is one of the most promising and safe measures in this respect. Biological control agents (BCAs) are considered to be more durable, more effective, and without any chemical residues affecting the human food chain (Choudhary and Johri, 2009). For example, non-pathogenic isolates of *F. oxysporum*, which were isolated from the crowns of healthy strawberry plants, suppressed the disease due to non-pathogenic growth in plants, thereby reducing the infection and/or the growth of the pathogen (Tezuka and Makino, 1991). However, the implementation of non-pathogenic isolates *F. oxysporum* as BCA is not widely accepted in strawberry farming systems. Therefore, an alternative source of BCAs is required. A limit for the introduction of BCAs to counteract soil-borne diseases is that they are poor colonizers on plants, so new attempts to improve biocontrol efforts have focused on the selection of microbes that can colonize the plant cells by the use of endophytes. Use of endophytic fungi as BCAs and the underlying mechanisms are now being studied extensively (Hyakumachi et al., 2014).

Dark septate endophytic (DSE) fungi represent a frequent root-associated fungal group that belong to the phylum Ascomycota (Jumpponen and Trappe, 1998), and colonize a great diversity of plant species (Mandyam and Jumpponen, 2005). Several DSE fungi have been used against fungal diseases such as rice blast disease caused by *Magnaporthe oryzae*, cabbage Verticillium wilt caused by *Verticillium longisporum*, and tomato wilt caused by *Verticillium dahliae* (Narisawa et al., 2004; Andrade-Linares et al., 2011; Su et al., 2013). Moreover, DSE fungi have been reported as plant growth promoters acting through enhancing nutrient acquisition of vegetable crops such as tomato, clover, and asparagus (Mahmoud and Narisawa, 2013; Della-Monica et al., 2015; Surono and Narisawa, 2017). However, these previous studies focused mainly on vegetable crops, and no studies have examined the potential of DSE fungi on fruit crops.

In this study, we aimed to investigate the effectiveness of DSE fungi to suppress Fusarium wilt with the ability to enhance the growth and fruit production of strawberry plants using artificial inoculation on soil systems. Therefore, we first investigated and conducted an inoculation experiment to evaluate disease suppression and the growth performance of strawberry plants in symbiosis with DSEs fungi.

## Materials and Methods

### Fungal Isolates

Fungal isolates were obtained from culture collection of Microbial Ecology Laboratory, Ibaraki University, Japan. These isolates were originally isolated from healthy roots of orchid plants *Spiranthes sinensis* and *Cyrtosia septentrionalis* in natural ecosystems in Shiga Prefecture (six isolates: SK46, SK47, SK48, SK50, SK51, and SK52), Okinawa Prefecture (four isolates: SK23, SK24, SK26, and SK31), and Ibaraki Prefecture (nine isolates: SK3, SK4, SK5, SK6, SK7, SK8, SK9, SK11, and SK12), Japan. Fungal isolates were recovered from glycerol culture stocks onto 50% corn meal (CM) agar medium (8.5 g CM agar, 15 g agar, and 1,000 mL distilled water). The identity of these nineteen fungal isolates are still unknown and will be molecularly identified for their taxa names and by phylogenetic analysis to estimate the relationships among taxa. The best candidates of DSE fungal isolates will be morphologically identified to support molecular datasets in this study.

### Preliminary Screening of Fungal Isolates for Their Pathogenicity

Pathogenicity screening was conducted to define non-pathogenic fungal isolates from pathogenic and other saprophytic fungi. Screening was conducted using a susceptible host plant Chinese cabbage (*Brassica campestris* L.) cv. Musou (Takii seed, Japan), family *Brassicaceae*, which is well known and can be used as a model plant for pathogenicity assays because of their sensitivity and fast response to the introduction of microbes. Fungal isolates were grown on oat meal agar (OMA) medium enriched with inorganic nutrients (1 g MgSO_4_.7H_2_O, 1.5 g KH_2_PO_4_, 1 g NaNO_3_, 18 g agar powder, and 1,000 mL distilled water) in Petri dishes (55 mm diameter), plates were incubated for 2 weeks at 23°C. The seeds of Chinese cabbage were surface sterilized by soaking in 70% ethanol solution for 1 min and then sodium hypochlorite (1% available chlorine) solution for 5 min, rinsed three times in sterilized distilled water (SDW), dried on sterile filter paper, and placed on 1.5% water agar culture medium (15 g agar, and 1,000 mL distilled water) for germination. After 2 days, the axenically grown seedlings were transplanted onto each of 19 growing fungal colonies on OMA medium. Plants transplanted onto non-inoculated fungi medium were used as control plants, and the whole set placed into sterile culture pots (CB-1, As One, Japan) and incubated in a growth chamber at 23°C under a light – dark cycle (18 h:6 h) for 2 weeks. Each treatment consisted of three pot replicates, with three individual plants per pot. The pathogenicity symptoms were observed and scored after 2 weeks incubation, as described by Narisawa et al. (2000). Plants were harvested and oven-dried at 40°C for 72 h to measure shoot dry mass (SDM). Plants with no visible symptoms (index 0) and exhibited SDM greater than or equal to control plants were defined and selected as potentially non-pathogenic DSE fungi candidates for the subsequent assay.

### DNA Extraction, Sequencing, and Phylogenetic Analysis

Fungal isolates were grown on 50% CM malt yeast (CMMY) culture medium (8.5 g CM agar, 15 g agar, 10 g malt extract, 1 g yeast extract, and 1,000 mL distilled water) incubated at 23°C for 2 weeks. Fresh mycelia were harvested and genomic DNA was extracted using PREPMAN Ultra Sample Preparation Reagent (Applied Biosystems, United States) in accordance with the manufacturer’s protocol. Polymerase chain reaction (PCR) was conducted to amplify the partial 18S small subunit (SSU), internal transcribed spacer (ITS) 1–5.8S-ITS2 regions, and partial 28S large subunit (LSU) region with universal primers ITS 5F and ITS 3F (White et al., 1990), and LR0R F and LR5 R (Vilgalys and Hester, 1990). PCR amplification was carried out in a 50 μL reaction mixture containing 100 ng of fungal genomic DNA, 0.2 μM concentration of each primer, 0.2 mM of each deoxy nucleoside triphosphate (dNTP), 10× Ex *Taq* buffer, 0.25 U of Ex *Taq* DNA polymerase (Takara Bio, Japan), and sterilized MilliQ water. PCR was carried out using a Takara PCR Thermal Cycler Dice (Takara Bio INC., model TP 600, Japan) with cycling conditions of 94°C for 4 min, 35 cycles of 94°C for 35 s, 52°C for 55 s, and 72°C for 2 min, followed by a final 10 min extension at 72°C. Following cycling, we used two methods for purification/clean up prior to running on an ABI sequencer instrument. PCR products were purified using 3 M sodium acetate (pH 4.8) 12 μL; 40% PEG 30 μL; 200 mM MgCl_2_ 1.5 μL; and 50 μL PCR product. PCR for sequencing was carried out in 10 μL reaction mixture containing 0.32 μL of each primer (10 μM), 1.5 μl of 5× sequencing buffer, 1.0 μl of primer (10 μM), 0.5 μL BigDye, 6.68 μL of sterilized MilliQ water, and 1.0 μL of purified DNA. The cycling conditions were: 96°C for 2 min, 25 cycles of 96°C for 30 s, 50°C for 15 s, and 60°C for 3 min. PCR sequencing products were purified using 3× volume of purification solution for PCR products as described previously. PCR sequencing products were resuspended in 20 μL Hi-Di^TM^ formamide solution (Applied Biosystems, United States) and sequence analysis was carried out using a BigDye Terminator v3.1 DNA sequencer instrument. The sequences obtained from each sequencing primer were assembled to a contig (1,200–1,500 bp) using ChromasPro software version 2.1.8 (Technelysium Pty Ltd.). The nucleotide sequences from three selected DSE isolates (SK47, SK48, and SK51) were deposited in the NCBI GenBank database under accession numbers MN811693–MN811695.

The sequences of DSE fungi were aligned with the sequences from GenBank using the basic local alignment search tool for nucleotides (BLASTN) program to find sequence homology with closely related taxa (Altschul et al., 1990). When the top three matching BLAST hits were from the same species and were ≥98% similar to the query sequence, this species name was assigned to the selected isolate. Phylogenetic analysis of molecular datasets from 19 fungal isolates in this study and similar sequences obtained from the GenBank database (Supplementary Table S1) were constructed and edited using MEGA-X (Kumar et al., 2018). The neighbor-joining method (Saitou and Nei, 1987) was used to infer the evolutionary history of the DSE fungal isolates, and bootstrapping was carried out using 1,000 replications to construct and assess the most stable phylogenetic relationships among taxa.

### Efficacy of DSE to Suppress *Fusarium oxysporum* f. sp. *fragariae* in Axenic Culture

In this experiment we screened six selected non-pathogenic DSE fungal isolates to suppress Fusarium wilt disease of strawberry plants as a target host plant. Fungal pathogen, Fof MAFF 744009 (GenBank NARO, Japan) was grown on potato dextrose agar (PDA) medium for 1 week. Six selected DSE fungi were inoculated into inorganic OMA medium. Strawberry (*Fragaria vesca* cv. Misaki (Sakata seed, Yokohama, Japan) seeds were surface sterilized by soaking in 70% ethanol solution for 1 min 30 s, and sodium hypochlorite (1% available chlorine) solution for 1 min. Seeds then were rinsed three times with SDW, dried overnight, and then placed on 1.5% water agar medium and incubated at 23°C for germination. Three-week-old strawberry seedlings were axenically transplanted onto each DSE fungal isolate. Seedlings transplanted onto un-inoculated DSE medium were used as control plants. The transplants were placed into sterile culture pots and incubated in a growth chamber at 23°C for 4 weeks in the same conditions. Three pot replicates were used for each treatment (each pot contained two seedlings), including the control plants. Fungal pathogen Fof was prepared by inoculation into water agar medium and incubated for 7 days at 23°C. Strawberry seedlings grown for 4 weeks with and without individual tested DSE fungal were challenged with Fof. The seedlings were overlaid together with the agar medium directly on top of Fof colonies, and incubation continued in the same conditions. Wilt disease symptoms were evaluated according to an index varying from 0 to 3 (with 0: no visible symptom; 1: slightly yellowing or stunted; 2: moderate yellowing or stunted, usually confined to slightly wilting; 3: severe wilting or death). Percentage of disease reduction was calculated using the formula described by Narisawa et al. (2000):

$$\mathrm{Percentage}\,\mathrm{Disease}\,\mathrm{Reduction}=100-\left[\left(\frac{A}{B}\right)\times 100\right]$$

where, A is the DSE inoculated value for disease index, B is the control value, aboveground (shoot biomass) of the plants was harvested and oven-dried at 40°C for 72 h. The SDM was measured and compared with control plants.

### Morphological Identification of Selected DSE Isolates

Pure fungal culture of three selected DSE isolates was grown on OMA medium at 23°C for 2 weeks. Small pieces (approximately 2 mm × 2 mm × 2 mm) of Pablum agar (Mead Johnson mixed Pablum; Canadian Post Corporation, Ontario, ON, Canada, 25 g; agar, 5 g; distilled water, 250 mL) were sandwiched between two 18 mm × 18-mm cover glasses (Matsunami Glass, Osaka, Japan), placed in a 9-cm water agar plate to provide humidity, and then incubated at 23°C. After 4 weeks, when the culture had grown sufficiently, Pablum agar was carefully removed and the cover glasses were appropriately mounted on 76 mm × 26 mm micro slide glasses using PVLG (16.6 g Polyvinyl alcohol, 100 mL lactic acid, 10 mL glycerin, and 100 mL ultrapure water). Conidiogenous cells and conidia were observed using a light microscope (BX51; Olympus, Tokyo, Japan).

### Effectiveness of the Selected DSE Isolates to Suppress Fusarium Wilt Disease Using Artificial Inoculation on Soil System

#### Inoculum Preparation and Mass Production

Three selected DSE fungi (SK47, SK48, and SK51) were grown in a 500 mL flask containing 250 mL of 2% malt extract broth (MEB) medium for 4 weeks at 23°C with 120 rpm shaking. Mycelia were harvested by filtering and washing with SDW until the liquid became clear to avoid transfer of any material from the MEB medium to the inoculum. Then, the mycelia were mixed with SDW for 1 min at minimum speed using an operating mixer at laminar flow to prevent contamination. The viability of each isolate was verified by plating the mycelium on 50% CMMY medium and incubated at 23°C. Mass production was made by transfer of 10 mL of inoculum (1 × 10^6^ hyphal fragments/mL) into a sterile plastic bag contained sterilized inoculant material (100 g wheat bran, 100 g rice bran, 300 g fermented leaves, and 340 mL distilled water). the inoculant was incubated in a growth chamber for 3–4 weeks and ready to mix with soil in the next experiment.

#### Soil Preparation for Strawberry Cultivation

Commercial inorganic soil (pH 6.0) was sterilized twice by autoclaving at 121°C for 30 min, 1 × 24 h. Two experiments were carried out in parallel consisting of strawberry plants grown in soil infested with Fof and non-infested soil.

#### Application of DSE Fungi Inoculum

Strawberry plants were obtained by seeds propagation and surface sterilized as described in the previous experiment. Soil was placed in pot (6 cm in diameter) and three-leaf-stage strawberry seedlings were inoculated with DSEs SK47, SK48, and SK51 with eight individual plants as replicates. Each inoculum of DSE isolates were mixed at 10% (w/w) into soil. Eight weeks after DSE fungi inoculation, the DSE-inoculated plants (DSE plants) and non-inoculated control plants were challenged with Fof.

#### Inoculation With Fof

Fof was grown on potato dextrose broth (PDB) culture medium at 23°C with 120 rpm shaking for 7 days, and the conidia were harvested. The conidial suspension was sieved using a Millipore filter with pore diameter 45 μm and its concentration adjusted to 1 × 10^6^ conidia/mL. Each pot was then inoculated by pouring 10% (v/w) of the conidial suspension onto the soil 8 weeks after DSE fungi inoculation. Cultivation was continued for all plants in a growth chamber at 23°C for 12 weeks.

#### Evaluation of Disease Severity and Plant Growth Parameters

The symptoms of Fusarium wilt were categorized as described previously. Disease severity was scored at the end of experiments for individual plants and visual severity ratings taken 12 weeks post-inoculation. At the end of the experiment, plants were harvested and plant growth parameters were calculated. SDM and root dry mass (RDM) was weighed after drying at 40°C for 72 h. Days to flower bud initiation and the number of fruits (NFRs) were calculated. Chlorophyll content was measured using a hand-held chlorophyll meter (SPAD-502; Minolta Camera Co., Osaka, Japan).

#### DSE Root Colonization Observations

Root colonization by DSE fungal isolates was observed to confirm whether the selected DSE fungi colonized the inner roots endophytically. Roots were harvested from plants after 150 days of cultivation. Root systems were washed thoroughly under a running tap water to remove adhering soil debris, then rinsed three times with SDW and used for root staining. The root samples were cleared with 10% (v/v) potassium hydroxide in a water bath at 80°C for 20 min. Subsequently, roots were acidified with 1% hydrochloric acid at room temperature for 5 min, then by staining with 50% acetic acid solution containing 0.005% cotton blue at room temperature (approximately 23°C), overnight. Root fragments (approximately 3 cm) were placed on slide glass and covered with a cover glass. Fungal colonization was observed using a light microscope (BX51; Olympus, Tokyo, Japan) equipped with Olympus DP25 digital camera.

### Statistical Analysis

All datasets in this study were calculated and analyzed statistically using one-way analysis of variance (ANOVA) and Tukey’s honestly significant difference test at *p* < 0.05 using SPSS version 20.0 (SPSS, IBM, Armonk, NY, United States).

## Results

Among nineteen fungal isolates, six fungal isolates (SK50, SK52, SK8, SK48, SK51, and SK47) showed no disease symptoms (disease index 0) on Chinese cabbage plants (Table 1). Moreover, there were no negative effects shown by no significant differences between the SDM of plants treated with these isolates and the control plants (Table 1). Thus, these non-pathogenic fungal isolates were used in the next experiment. Thirteen other fungal isolates (SK31, SK46, SK24, SK12, SK6, SK9, SK11, SK26, SK3, SK4, SK5, SK23, and SK7) showed disease symptoms and exhibited disease index values from 1 to 3. Therefore, these isolates were excluded from further experiments (Table 1).

**TABLE 1:** The effect of fungal isolates on the shoot dry mass and disease index of Chinese cabbage^1^.

DSE isolates	Disease index	Shoot dry mass (mg) mean ± SE
SK7	3	2.45 ± 0.35^a^
SK23	3	2.46 ± 0.25^a^
SK5	3	2.46 ± 0.78^a^
SK4	3	3.67 ± 0.05^abc^
SK3	3	3.99 ± 0.30^abc^
SK26	1	4.60 ± 1.42^abcd^
SK11	2	4.76 ± 0.97^abcd^
SK9	2	5.25 ± 1.21^abcde^
SK6	1	5.47 ± 0.74^bcde^
SK12	2	5.49 ± 0.49^bcde^
SK24	1	5.49 ± 0.88^bcde^
SK46	2	5.92 ± 0.71^cdef^
SK47	0	6.34 ± 1.57^cdef^
SK51	0	6.36 ± 1.26^cdef^
SK48	0	6.37 ± 1.53^cdef^
SK8	0	6.61 ± 0.73^cdef^
SK31	2	6.89 ± 1.54^def^
SK52	0	7.96 ± 1.12^ef^
SK50	0	8.51 ± 0.79^f^
Control	0	6.67 ± 0.56^cdef^

*^1^Means followed by the same letter in column are not significantly different at *p* < 0.05 according to Tukey’s honestly significant difference test (*n* = 3).*

The highly conserved SSU, ITS, and LSU nrRNA regions (1,200–11,500 bp) of fungal isolates were sequenced and subjected to phylogenetic analysis. Homology searches using the BLASTN program at GenBank NCBI are shown in Table 2. Based on molecular identification, the majority of fungal isolates used in this study included DSE species of *Exophiala* and *Cladophialophora*, accounting for 26.32% (5/19) (Table 2). This indicates that *Exophiala* and *Cladophialophora* are dominant in areas of the sampling sites.

**TABLE 2:** Best BLAST matches for DSE fungi based on SSU, ITS, and LSU regions.

Isolates number	Fungal taxon	Sequence similarity (%)	Accession number of SSU + ITS + LSU in NCBI
SK3	*Cadophora malorum*	99	MF494620
SK4	*Cadophora malorum*	99	MF494620
SK5	*Phialocephala* sp.	91	LN813032
SK6	*Cladophialophora* sp.	98	LC192126
SK7	Uncultured *Phialocephala*	98	KF660561
SK8	*Exophiala pisciphila*	98	AF050272
SK9	*Scolecobasidium* sp.	99	HQ607847
SK11	*Scolecobasidium* sp.	100	HQ607847
SK12	*Cladophialophora* sp.	94	LC192126
SK23	*Helicoma isiola*	96	EF010926
SK24	*Scolecobasidium* sp.	98	KU529844
SK26	*Scolecobasidium* sp.	100	KU529844
SK31	*Exophiala* sp.	94	AB488490
SK46	*Exophiala* sp.	94	AB488490
SK47	*Exophiala* sp.	95	AB488490
SK48	*Exophiala pisciphila*	98	AF050272
SK50	*Cladophialophora chaetospira*	99	EU035403
SK51	*Cladophialophora chaetospira*	99	EU035405
SK52	*Cladophialophora chaetospira*	99	EU035406

**FIGURE 1:**
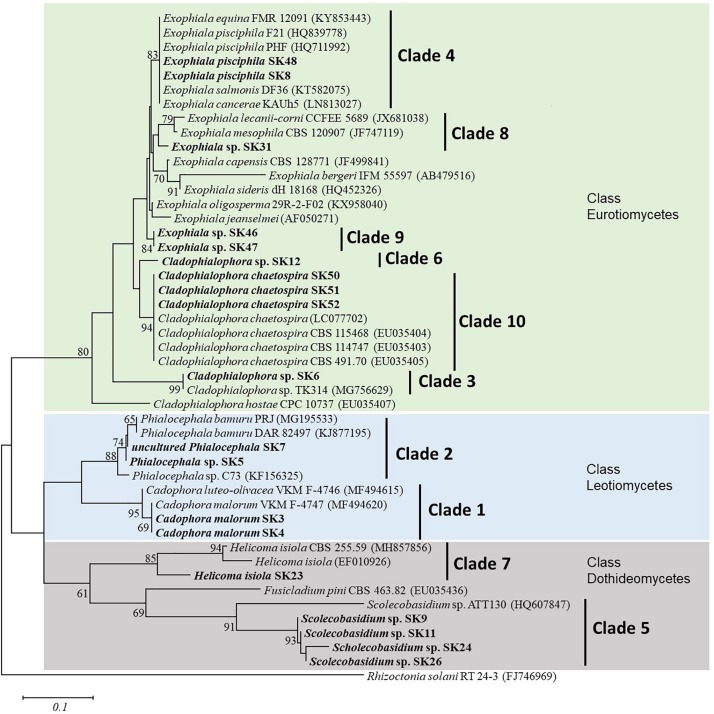
Neighbor-joining phylogenetic tree of nineteen fungal isolates in the present study and reference sequences retrieved from the NCBI GenBank database. This tree was derived from the analysis of three (SSU, ITS, and LSU) sequences. *Rhizoctonia solani* RT 24-3 (FJ746969) was used as an outgroup. Numbers at the nodes are bootstrap support values (percentage of 1,000 replicates); only values above 60% are displayed. *Bar* represent 0.1 substitutions per nucleotide position. The fungal isolates from this study are in bold.

Phylogenetic analysis of SSU, ITS, and LSU nrRNA sequences were constructed to study the relationships among taxa (Figure 1). The sequences of the 19 fungal isolates were clustered into three classes of Ascomycota and consisted of 10 clades. As evidenced by the phylogenetic analysis, isolates SK46 and SK47 were in a separate clade from the other *Exophiala* species and may represent a novel species (Figure 1). The selected non-pathogenic fungal isolates formed distinct lineages and clustered into clades 4, 9, and 10.

**FIGURE 2:**
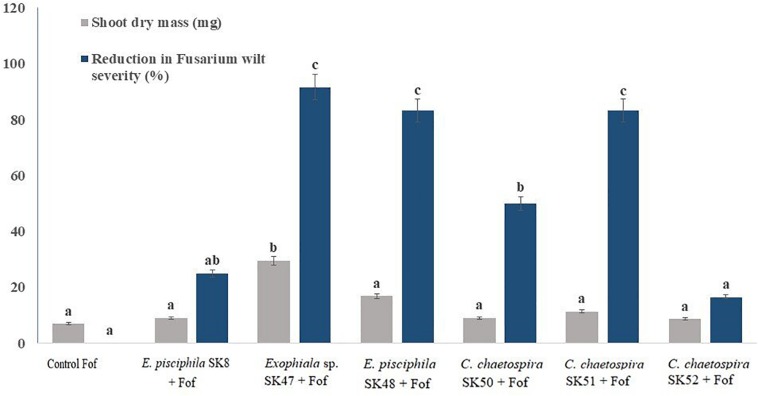
Efficiency of *E. pisciphila* SK8, *Exophiala* sp. SK47, *E. pisciphila* SK48, *C. chaetospira* SK50, *C. chaetospira* SK51, and *C. chaetospira* SK52 in suppressing Fusarium wilt. Bars with the same lowercase letter are not significantly different according to Tukey’s honestly significant difference test at *p* < 0.05. Error bars represent standard error of mean (*n* = 3).

In axenic culture pots *Exophiala* sp. SK47 was the most effective and suppressed plant disease by 91.67%. It was followed by *E. pisciphila* SK48 and *C. chaetospira* SK51 that reduced disease of 83.33% (Figure 2), while *E. pisciphila* SK8, *C. chaetospira* SK50, and SK52 reduced Fusarium wilt by 50% (Figure 2). Therefore, based on the ability of *Exophiala* sp. SK47, *E. pisciphila* SK48, and *C. chaetospira* SK51 to reduce Fusarium wilt disease by >50%, these DSE fungi were selected for the next experiments using soil systems.

The three selected DSE isolates were used for morphological and molecular identification. *Exophiala* sp. SK47 was hyaline to light brown, with septated hyphae, and brown colony color on 50% CMMY agar medium. Conidial cell formation had not occurred after 8 weeks, incubation at 23°C. Therefore, we were unable to confirm its identity at a species level. Based on our phylogenetic tree analysis (Figure 1), strong bootstrap support (80%) within *Exophiala* isolates confirmed their congeneric taxonomy and close affinity to the *Exophiala* genus. Interestingly, this isolate formed a different clade (clade 9) with another strain in the phylogenetic tree (Figure 1), which may represent a novel species. DSE isolates SK48 and SK51 were identified both morphologically and molecularly as *E. pisciphila* and *C. chaetospira*, respectively. *E. pisciphila* SK48 was formed an annelidic conidiogenesis producing slimy heads of conidia with conidia 3–6 μm in length, smooth with no septate, and clusters at the tip of conidiogenous cells. Colonies of *C. chaetospira* SK51 was olivaceous-black on 50% CMMY agar medium, and margins smooth. The hyphae were giving rise to conidiophores with catenulate conidia. Conidiophores with septate conidia (length 14–29 μm), conidia are fusiform and spindle-shaped. Conidia in branched, acropetal chains, and remain attached in long chains, as described by Crous et al. (2007).

**FIGURE 3:**
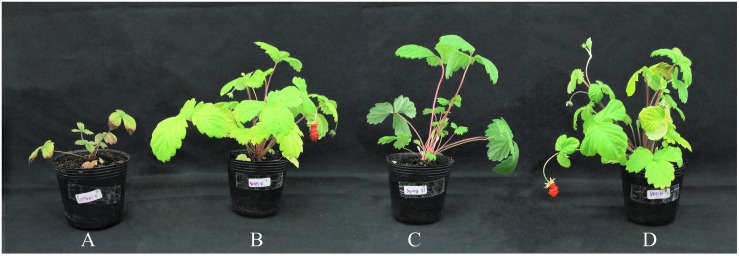
Performance of strawberry plants inoculated with DSE fungal isolates under Fof challenge in inorganic commercial soil **(A)** Control plants. **(B)** Plant treated with *Exophiala* sp. SK47. **(C)** Plant treated with *E. pisciphila* SK48. **(D)** Plant treated with *C. chaetospira* SK51. All plants were infested with Fof, including the control plant.

**FIGURE 4:**
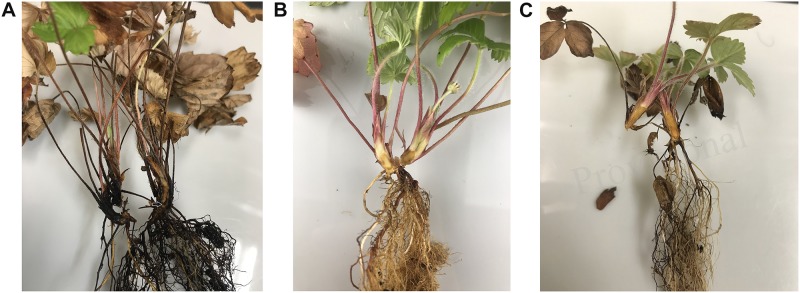
Strawberry crowns cut longitudinally; exposing vascular tissue discoloration (shown by arrow). **(A)** Infested Fof and non-treated DSE (positive control). **(B)** Non-infested Fof and non-treated DSE (negative control). **(C)** Infested Fof, treated DSE.

Application of three selected DSE fungal isolates to suppress Fusarium wilt disease was conducted as two experiments, i.e., soil-infested Fof and soil non-infested Fof, to observe the performance of strawberry plants in association with selected DSE fungi. Strawberry Fusarium wilt symptoms characterized by stunting of younger leaves, lesions on the petioles, rapid yellowing, and wilting of the plant (Figure 3). Cross sections of the crown of plant infected by Fof is shown by the dark color of roots, brown or black discoloration of the vascular tissue, and by crown rot (Figure 4). Fof was most frequently isolated from crowns and was the dominant pathogen associated with crown discoloration of strawberry. This pathogen was isolated from infected crowns and roots (Fang et al., 2012).

The rate of disease incidence in plants varied among treatments (Table 3). *C. chaetospira* SK51-treated plants scored the highest disease severity reduction ratings at 90.48%, with a chlorophyll content significantly different from control plants under Fof challenge (Table 3). *Exophiala* sp. SK47-treated plants exhibited disease severity reduction of 85.71%, followed by *E. pisciphila* SK48-inoculated plants at 61.90%. DSE fungi in this study were found to promote plant growth through increased photosynthetic rate, since inoculating strawberry plants with DSE fungi led to an increased chlorophyll content and biomass of SDM and RDM (Table 3).

**TABLE 3:** Effectiveness of DSE fungal isolates on disease reduction, chlorophyll content, growth parameters: shoot dry mass (SDM), root dry mass (RDM), beginning of flowering and fruiting, and number of fruit (NFR) at the end of experiments^1^.

Experiment	Tested DSE	Disease reduction (%)	Chlorophyll content (SPAD)	SDM (mg plant^–1^)	RDM (mg plant^–1^)	Flower bud initiation (days)	NFR
Inorganic soil	Control	0.00a	18.70a	213.00a	77.51a	150	0.00a
infested	*Exophiala* sp. SK47	85.71c	26.68ab	1,764.10c	751.97b	50	7.37c
Fusarium	*E. pisciphila* SK48	61.90b	25.60ab	1,077.10b	330.18a	110	2.75b
	*C. chaetospira* SK51	90.48c	31.85b	1,802.88c	618.30b	40	7.62c
Inorganic soil	Control	NA	NA	219.88a	159.53a	126	0.62a
non-infested	*Exophiala* sp. SK47	NA	NA	1,724.92c	699.50b	43	6.37b
Fusarium	*E. pisciphila* SK48	NA	NA	1,028.20b	824.97b	90	3.00a
	*C. chaetospira* SK51	NA	NA	2,328.08c	1,357.40c	37	9.25c

*^1^Means followed by same letter in same column not significantly different at *p* < 0.05 according to Tukey’s least significant different test (*n* = 8). NA, not applicable.*

**FIGURE 5:**
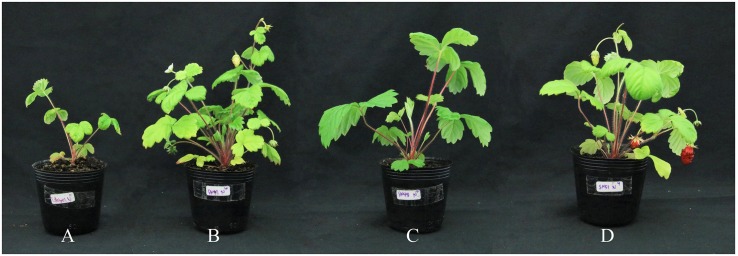
Performance of strawberry plants inoculated with DSE fungal isolates in inorganic commercial soil. **(A)** Control plant. **(B)** *Exophiala* sp. K47-treated plant. **(C)** *E. pisciphila* SK48-treated plant. **(D)** *C. chaetospira* SK51-treated plant.

In soil non-infested with Fof, the performance of strawberry plants treated with the three selected DSE fungi showed consistent results with soil infested Fof in SDM, RDM, and NFR (Figure 5 and Table 3). These results suggested that flower bud initiation and fruit formation may be affected by interactions between DSE and strawberry plants.

**FIGURE 6:**
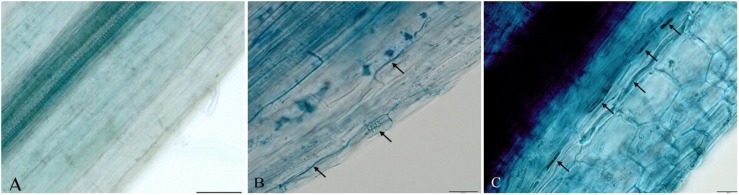
Melanized fungal hyphae formed by DSE fungi in strawberry roots. **(A)** Non-treated DSE as control roots. **(B)** *Exophiala* sp. SK47-treated roots. **(C)** *C. chaetospira* SK51-treated roots. Bars: 50 μm **(A)**, 20 μm **(B–C)**.

In order to determine the endophytism of DSE isolates in strawberry roots, we observed fungal structures (Figure 6). The occurrence of blue stained hyphae (melanized hyphae) in roots was observed (Figure 6). Ubiquitous distribution of fungal structures from *E. pisciphila* SK47 and *C. chaetospira* SK51 in the roots confirmed the endophytism of these isolates in strawberry roots, conferring some beneficial traits, i.e., Fof tolerance, acceleration of flower initiation, and fruit formation.

## Discussion

In response to disease, the symbiotic association of endophytic fungi with host plants can reduce the adverse effects of stress. DSE fungi are reported to be able to associate with a variety of agricultural and forest plants, but there are no reports of a favorable symbiotic relationship between DSE fungi and strawberry plants, especially of an association to increase the resistance of strawberry plants against Fof as well as growth promotion and fruit production in cultivated strawberry. In this study, we screened 19 fungal isolates for their pathogenicity and growth-promoting effects. Six isolates determined as being non-pathogenic were examined further for their function to controls Fof. The six selected fungal isolates were molecularly identified as *E. pisciphila* SK8, *Exophiala* sp. SK47, *E. pisciphila* SK48, and *C. chaetospira*: SK50, SK51, and SK52.

In strawberries, Fof invades water-conducting tissues of the crown and leaves, thus blocking water and nutrients supply (Paynter et al., 2014). This infection causes plant yellowing, wilting, stunting, and eventually death. Due to the infection site of Fof, this research selected root-colonizer DSE fungi to overcome Fof infection by enhancing plant health and production either in the presence or absence of Fof in soil. Our observation of DSE fungus *C. chaetospira* SK51 colonization showed that this fungus heavily colonized plant roots inter- and intracellularly (Figure 6). This indicated that the colonization of this fungus was able to occupy root plant niches in competition with Fof resulting in disease severity reduction and increased plant growth and production. In contrast, plants infested with Fof and non-treated DSE showed plant death (Figures 3, 4) due to the growth and colonization of Fof in epidermal cells, the cortex, and entering the xylem vessels, spreading to colonize and ultimately block the vascular system, causing the plant to wilt and eventually death (Lagopodi et al., 2002; Xiao-min et al., 2011).

*Exophiala* sp. isolate SK47 was not successfully identified to the species level based on molecular (SSU, ITS, and LSU sequences) and morphological characteristics in our study. BLASTN results with GenBank showed 95% similarity with *Exophiala* sp. Phylogenetic analysis revealed that *Exophiala* comprise species complexes (Figure 1). Phylogenetic analysis supported the distinct lineage of isolate SK47 from *E. equina*, *E. pisciphila*, *E. salmonis*, *E. cancerae*, *E. lecanii-corni*, *E. mesophila*, *E. capensis*, *E. bergeri*, *E. sideris*, *E. oligosperma*, and *E. jeanselmei*, indicating this isolate might represent a novel species within *Exophiala* (Figure 1). Further analysis is necessary for the identification at the species level, such as analysis of translation elongation factor 1-α, β-tubulin, and actin genes, and analysis of secondary metabolites. However, most studies on *Exophiala* species have focused on their importance as etiologic agents of diseases in animals and humans (Richards et al., 1978; Zeng and de Hoog, 2008; De Hoog et al., 2011; Najafzadeh et al., 2013; Wen et al., 2015). Because we were not able to identify the species for *Exophiala* sp. isolate SK47, thus we were unable to clarify its pathogenicity in animals and humans. Therefore, we excluded this isolate and we will assess its identification at a species level and its role in future research.

*Cladophialophora chaetospira* SK51 was successfully identified molecularly and morphologically in this study. Strawberry plants treated with *C. chaetospira* SK51 had the highest RDM and NFR values both in infested and non-infested Fof. DSE fungus *C. chaetospira* SK51 suppressed disease severity by 90.48%, and its chlorophyll content was significantly different between control and other DSE-treated plants (Table 3). The increase in chlorophyll content indicated that the photosynthetic rate was increased and led to plant health, growth promotion, and fruit production, as shown by the growth parameters measured in this study (Table 3). Chlorophyll content (SPAD-502) was highly correlated with photosynthetic rate as well as the leaf nitrogen and potassium concentrations (Sim et al., 2015). Nitrogen plays a pivotal role in the inorganic nutrition of plants, determining growth (Mengel, 1994; Marschner, 1997) and as a major component of chlorophyll (Wang et al., 2004). Therefore, we suggested that application of *C. chaetospira* SK51 effectively promoted strawberry plant growth and fitness by facilitating nutrient uptake of nitrogen and potassium from soil. Our results showed that one fungal isolate can have multiple benefits for host plants. *C. chaetospira* syn. *Heteroconium chaetospira* (Grove) M. B. Ellis has been reported to suppress clubroot disease in Chinese cabbage plants (Narisawa et al., 2005). Lahlali et al. (2014) reported that *Heteroconium chaetospira* (syn. *C. chaetospira*) suppressed clubroot (*Plasmodiophora brassicae*) disease via the upregulation of host genes involved in jasmonic acid, ethylene, and auxin biosynthesis. The result indicated that these genes may also be involved in inducing resistance in canola by *H. chaetospira* against clubroot.

Some of BCAs have shown efficacy against *F. oxysporum* from diverse plant crops, in controlled experimental conditions *in vitro* and *in planta. Trichoderma virens* and *Trichoderma viride* significantly increased the amount/activity of secreted antifungal metabolites *in vitro*, response to volatile compounds produced by seven strains of f. sp.: *radices-lycopersici* (eggplant), *lycopersici* (tomato), *ciceris* (chickpea), *conglutinans* (cabbage), *pisi* (pea), *cubense* (banana), and *melonis* (melon) (Li et al., 2018). In field conditions, Fusarium wilt of banana has been controlled up to 79% using *Pseudomonas* spp. strains, and up to 70% by several endophytes and *Trichoderma* spp. strains (Bubici et al., 2019). *Trichoderma harzianum* showed *in vitro* inhibition of mycelial growth of *F. oxysporum* f. sp. *ciceris* (Kumar and Mane, 2017). Possible modes of action of BCAs may involve direct antagonism (e.g., antibiosis) against *F. oxysporum* f. sp. *cubense* and can affect the plant physiology and/or the microbiota with consequent, indirect effects against the pathogen. Induction of plant local/systemic resistance, and plant growth promotion are typical mechanisms that indirectly act against the pathogen, or at least contribute to reducing the infections or the disease (Bubici et al., 2019). Plant growth-promoting rhizobacteria isolates of *Pseudomonas* and *Bacillus* were evaluated for their volatile compound (peroxidase and polyphenol oxidase) efficacy against mycelial growth of *F. oxysporum* f. sp. *melongenae* on eggplant *in vitro* (Altinok et al., 2014). Studies on BCAs have been mostly limited to *in vitro* conditions so far, with very few pot experiments, and none conducted in the field.

We demonstrated that root-colonizer DSE fungus *C. chaetospira* SK51 was a remarkable BCA as well as a growth promoter in strawberry plants. Roots of strawberry plants are exposed to various soil-borne pathogens. Therefore, it may be an effective strategy to protect plant roots using root-colonizer DSE fungi. Furthermore, roots have important roles: (1) anchoring the plant, (2) water absorption, and (3) nutrient uptake from soil, which are necessary for growth and fruit production.

This study provides a new insight that DSE fungus *C. chaetospira* SK51 is symbiotic with strawberry plants and effectively stimulates plant health and increases photosynthesis rate, thereby triggering flower bud initiation and fruit production. The mechanisms by which this DSE fungus improved resistance and plant growth remain unknown, but the effectiveness of this fungus in soil systems indicates that *C. chaetospira* SK51 has potential as a new BCA with growth-promotion functions. Further investigations related to the mode of action, pathogenesis-related proteins, induced systemic resistance, and flowering-related genes will be conducted.

## Conclusion

In the present study, we screened and investigated the effectiveness of DSE fungal isolates in suppressing strawberry Fusarium wilt disease and on growth promotion activities. The DSE fungus *C. chaetospira* SK51 act as a BCA as well as a growth promoter, over control plants and plants treated with other DSE isolates. Root colonization by this fungus was visualized as evidence that root-associated fungus *C. chaetospira* SK51 conferred plant protection and improved plant health by enhancing growth parameters. Agricultural application of *C. chaetospira* SK51 in soil environment may be a promising strategy to suppress Fusarium wilt during nursery to field production, and we demonstrated that *C. chaetospira* SK51 has an ability to naturally colonize plant root tissues and establish beneficial traits in strawberry plants. The mechanism of action of *C. chaetospira* SK51 in modulating plant disease tolerance via upregulating defense-related proteins is a topic for future research.

## Data Availability Statement

Publicly available datasets were analyzed in this study. This data can be found here: MN811693, MN811694, MN811695.

## Author Contributions

KN supervised the research and secured the funding. KN and WH planned and designed the research, analyzed and interpreted the data. WH performed the experiments, constructed the phylogenetic analysis, deposited the DNA sequences to the GenBank, and wrote the manuscript. All authors critically reviewed and edited the manuscript.

## Conflict of Interest

The authors declare that the research was conducted in the absence of any commercial or financial relationships that could be construed as a potential conflict of interest.
