# Supplementary material for: The Effectiveness of a Dark Septate Endophytic Fungus, Cladophialophora chaetospira SK51, to Mitigate Strawberry Fusarium Wilt Disease and With Growth Promotion Activities
Source: Front Microbiol. 2020 Apr 15;11:585. doi: 10.3389/fmicb.2020.00585 (PMC7174500; doi:10.3389/fmicb.2020.00585)
Supplement: Supplementary file 1 [file Table_1.DOCX]

Supplementary Material

# Supplementary Data

**TABLE S1** GenBank accession numbers of sequences used for phylogenetic analyses

| A | B | C | D |
| --- | --- | --- | --- |
| Genus | Species | Voucher/Strain | SSU+ITS+LSU |
| Cadophora | malorum | VKM F-4747 | MF494620 |
| Cadophora | luteo-olivacea | VKM F-4746 | MF494615 |
| Cladophialophora | chaetospira |  | LC077702 |
| Cladophialophora | chaetospira | CBS 115468 | EU035404 |
| Cladophialophora | chaetospira | CBS 114747 | EU035403 |
| Cladophialophora | sp. | TK314 | MG756629 |
| Cladophialophora | hostae | CPC 10737 | EU035407 |
| Cladophialophora | chaetospira | CBS 491.70 | EU035405 |
| Exophiala | salmonis | DF36 | KT582075 |
| Exophiala | pisciphila | PHF | HQ711992 |
| Exophiala | equina | FMR_12091 | KY853443 |
| Exophiala | jeanselmei |  | AF050271 |
| Exophiala | pisciphila | F21 | HQ839778 |
| Exophiala | lecanii-corni | CCFEE 5689 | JX681038 |
| Exophiala | bergeri | IFM 55597 | AB479516 |
| Exophiala | mesophila | CBS 120907 | JF747119 |
| Exophiala | sideris | dH 18168 | HQ452326 |
| Exophiala | capensis | CBS 128771 | JF499841 |
| Exophiala | cancerae | KAUh5 | LN813027 |
| Exophiala | oligosperma | 29R-2-F02 | KX958040 |
| Fusicladium | pini | CBS 463.82 | EU035436 |
| Helicoma | siola | CBS 255.59 | MH857856 |
| Helicoma | isiola |  | EF010926 |
| Phialocephala | sp. | C73 | KF156325 |
| Phialocephala | bamuru | PRJ | MG195533 |
| Phialocephala | bamuru | DAR 82497 | KJ877195 |
| Rhizoctonia | solani | RT 24-3 | FJ746969 |
| Scolecobasidium | sp. | TT130 | HQ607847 |
|  |  |  |  |
